# Improving management of tuberculosis in people living with HIV in South Africa through integration of HIV and tuberculosis services: a proof of concept study

**DOI:** 10.1186/s12913-018-3524-9

**Published:** 2018-09-14

**Authors:** Irit Sinai, Farley Cleghorn, Hans Friedemann Kinkel

**Affiliations:** 1 Palladium, 1331 Pennsylvania Ave., NW, Washington, DC 20004 USA; 2 Department of Family Medicine, University of Pretoria, Pretoria, South Africa; 3 Department of Tropical Medicine and International Health, Charité – Universitätsmedizin Berlin, Berlin, Germany

**Keywords:** TB, HIV, Service integration, South Africa, Primary care

## Abstract

**Background:**

South Africa’s tuberculosis burden is the third highest globally and is closely associated with the country’s devastating HIV epidemic. The separation of HIV and TB services in primary healthcare services in South Africa hampers TB case finding in patients who are co-infected with HIV and TB. This operational proof of concept study assessed an approach to improving tuberculosis detection and treatment by integrating tuberculosis management into HIV care.

**Methods:**

The intervention involved workforce re-engineering accompanied by changes to the physical environment in three primary healthcare facilities in Gert Sibande district, Mpumalanga Province, that allowed HIV providers to test their HIV patients for TB and initiate and monitor TB treatment when indicated. To assess the proof of concept we compared the management of TB patients by HIV and TB providers, by reviewing the records of all facility patients who tested positive for tuberculosis between July 2015 and February 2016. We also considered the perceptions of healthcare providers and facility managers about the intervention through structured interviews.

**Results:**

Approximately 30% of the 1855 patients with presumed TB in the three clinics had been identified by HIV providers. The percentage of patients consecutively tested for TB was 81.0% and 85.0% (*p* = 0.0551) for HIV and TB providers, respectively. Of the patients identified with TB by HIV and TB providers, 75.4% and 79.2% (*p* = 0.2876), respectively, were initiated on treatment. The defaulter rate was higher among HIV, compared to TB, providers (12.8% versus 4.2%). Overall, healthcare providers and facility managers had positive views of the intervention but raised concerns regarding potential increase in workload and administrative issues, as well as infection control.

**Conclusions:**

The results of this proof-of-concept study indicate that the full spectrum of TB services can be easily and effectively integrated into existing HIV care programs. However, a possible shift in the service providers’ workload, including administrative tasks, must be tackled and effective infection control must be ensured. Further research is needed to assess the impact of TB service integration into the scope of HIV care (or other chronic care programs) on patient outcomes, including analysis of routine data.

**Electronic supplementary material:**

The online version of this article (10.1186/s12913-018-3524-9) contains supplementary material, which is available to authorized users.

## Background

One in six adults in South Africa is HIV positive [1, 2], and the country’s TB burden is the third highest in the world, with about 1% of the population of about 50 million developing active TB disease each year [3, 4]. Incidence of new TB cases has increased four-fold over the past 20 years, due primarily to the widespread HIV epidemic in the country [5, 6]. In response to the devastating HIV and TB epidemics, which were paralleled by alarmingly high maternal and child mortality rates, and an increase in chronic and non-communicable diseases, the South African government initiated a comprehensive health sector reform in 2009 [7, 8]. At the core of the reform was a 10-point plan that aimed at “[…] creating a well-functioning health system, capable of producing improved health outcomes.” [7]. Accelerated attainments in HIV/AIDS (“Combatting HIV and AIDS”) and TB (“Decreasing the burden of disease from tuberculosis”) were among the four key target areas of the strategic framework guiding this reform [7].

A review of the South African HIV and TB programs in 2014, guided by the World Health Organization (WHO) and the United Nations International Children’s Emergency Fund (UNICEF), revealed “impressive” progress [9]. This included the massive scale up of antiretroviral treatment (ART) services since 2009, a significant increase in the number of people on ART, the number of people tested for HIV, and the number of people screened for TB. In addition, TB case detection had markedly increased as did the number of facilities offering treatment for drug-resistant TB [9].

More recent data show that in 2016, 86% of people living with HIV in south Africa knew their status and that of the 7.1 million people eligible for ART, 3.9 million people were on ART [2]. In 2016 237,000 new (and relapsed) cases of TB were detected, and of those 96% had been tested for HIV and informed of the results. Nearly 60% of the people newly infected with TB were HIV positive [4]. ART coverage of HIV-positive TB patients was 88% in 2016, increasing from 54% in 2012 and 62% at the end of 2013 [10]. However, effective TB screening and testing of HIV patients remains a challenge [11], partly because to be effective, TB screening in people living with HIV must be performed in every clinic visit, and facilities should be set up in a way that allows for prompt TB testing, highlighting the need for effective integration of HIV and TB services [12].

This challenge has remained despite several promising developments that were initiated at about the same time. With the introduction of the GenXpert test in 2011 in South Africa for routine TB testing, following WHO recommendations, TB testing has become significantly quicker and more accurate at primary healthcare facilities [13]. However, while the 2014 national TB guidelines include a chapter on TB and HIV that directs TB providers to test their patients for HIV and initiate ART treatment for co-infected patients, the link between HIV and TB services is still understood only as desirable integration of services, rather than improved collaboration between the two services [13].

Similarly, the 2015 ART guidelines, which highlight the importance of TB screening of HIV patients, does not explicitly call for an integration of services [14]. Finally, the ‘Ideal Clinic’ initiative, which was introduced by the South African government in 2013, remains vague about the integration of TB and HIV [15–17]. The program strives for an ‘integration of clinical service management’ (ICSM) which means that chronic conditions (among them HIV and TB) are supposed to be managed by the one team. The program, however, does not explicitly guide the integration of HIV and TB services [15–17].

### Patient flow and service organization

In theory, five different models for integrating HIV and TB services can be distinguished: (1) entry via TB service, with referral for HIV testing and care; (2) entry via TB service, on-site HIV testing, and referral for HIV care; (3) entry via HIV service with referral for TB screening and treatment; (4) entry via HIV service, on-site TB screening, and referral for TB diagnosis and treatment; and (5) TB and HIV services provided at a single facility by a single team [18].

So far, in most public health facilities in South Africa HIV and TB services are provided according to models 2 or 3 [18]. For an HIV patient this means that s/he queues for HIV services. At the start of the HIV visit, the provider asks the HIV patient about TB symptoms. If symptomatic, i.e. responds “yes” to one or more of the questions about TB symptoms, the patient is referred to TB services for testing, often in the same facility. The patient then has to queue for the TB service, where s/he gets a sputum bottle and is instructed to go outside to collect sputum and return to the TB service. If the patient is diagnosed with TB, TB treatment is usually provided and monitored by the TB provider.

Similarly, a TB patient first has to queue for TB services. During the initial visit, s/he is asked about his/her HIV status. If s/he has never been tested, or the last negative test is older than 12 months, the patient is tested for HIV. This can be initiated by the TB provider but is often done by HIV counselling and testing providers at the same facility. If the patient is found to be HIV positive, s/he may be initiated on ART by the TB service provider or alternatively by the HIV provider, however, monitoring ART is done by HIV providers only.

There are several problems inherent in this practice. First, patients who are screened for TB symptoms and found to be symptomatic are inconvenienced by the need to wait for the TB service, having already waited in line to see the HIV provider when they arrived at the facility. Anecdotal evidence suggests that some patients lose their patience and leave the facility before they see the TB provider to obtain their sputum test [19]. If these patients have TB, this is a lost opportunity for early diagnosis and treatment. By the time they return to the facility they may already be very sick and may have spread the infection to others. This applies analogously also to TB patients who are found to be HIV positive.

Second, because of the vertical structure of services, TB providers do not, or only poorly, communicate test results of TB, or TB-treatment details, with HIV providers who treat the same patient for HIV. TB providers can and do test their patients for HIV, and if positive initiate treatment. However, HIV providers suspecting TB are usually not allowed to test for TB. They need to refer the patient to the TB service. If the patient should be treated for both HIV and TB, the two providers treat and follow up the two diseases separately. Patient records are also kept separately.

Studies show that such vertical organization of services can result in reduced TB case findings and poor or delayed linkage to care, as well as low rates of ART-initiation [20–22]. Better integration of TB and HIV services, so that they are offered through a coordinated approach, can address these shortcomings and result in more efficient and cost-effective services [23]. The fragmentation of HIV and TB services in primary healthcare facilities, therefore, remains a challenge [24]. A very recent call by Deputy Director-General at South Africa’s National Department of Health for integration of TB and HIV services is thus more relevant than ever [25].

### Objectives of the intervention

This proof-of-concept study investigated an approach for better integration of TB and HIV services. The intervention involved HIV health-care providers in TB testing and treatment of their HIV patients. The intervention was designed to:

1. Study the practicalities of integrated HIV and TB services at the provider level; and
2. Assess the effect of the integrated HIV and TB services on the outcome of TB treatment.

## Methods

This implementation science research is an operational proof-of-concept study, which tested an approach to HIV and TB service integration. Specifically, we studied an intervention that modified the typical patient flow described above, in such a way that HIV patients can be tested and treated for TB by their HIV health provider. The study established whether this modification is feasible and practical; whether it is acceptable to health personnel; and the potential of the intervention to improve health outcomes.

### Study settings

Three public health facilities were selected in Gert Sibande District, Mpumalanga province, which has one of the highest proportions of HIV and TB in South Africa [26] and which was listed in South Africa’s National Strategic Plan on HIV, TB, and STIs as one of 27 districts in the nation that contribute 82% of HIV cases [27]. The study facilities were selected based on patient volume (relatively high volume of TB and recently diagnosed HIV cases), and such that each facility utilized a different GeneXpert machine. In this way no one lab was overburdened by the study. No other studies and no significant renovations or operational changes were current or planned in the selected facilities. The facilities varied in size. Volume of patients on ART at the start of the study ranged from about 1100 to 2500. In the 3 months period prior to the start of the study (January-March 2014), the three facilities submitted 119, 179, and 204 GeneXpert tests respectively. One facility was open only during regular working hours five days a week; one provided services 24 h per day, including weekends; the third was open 24/7, but provided HIV and TB testing and treatment only during regular working hours. To protect confidentiality, we do not provide facility names but use numbers 1, 2 and 3 to identify the clinics instead.

### The intervention

The intervention, which ran from July 2015 to May 2016, consisted of training of facility staff, as well as minor infrastructure changes in the facility, to allow HIV providers to test their HIV patients for TB, initiate TB treatment as needed, and follow up their patients through recovery. All HIV-service nurses in the study facilities were trained to implement current procedures for TB testing and treatment, including infection control, using the training program which the district uses to train TB nurses (known as PC 101). The training was done by the Gert Sibande district TB trainers with support from the study coordinator. Staff from the South African National Health Laboratory Service (NHLS) trained on GeneXpert use. HIV providers were then instructed to not only screen their HIV patients for TB symptoms at every visit as they were already doing, but to also test their symptomatic patients for TB and treat patients with confirmed TB for the disease. Providers were also taught and encouraged to retrieve TB sputum test results directly from the NHLS database.

At the start of the intervention, the study coordinator worked with facility managers and with the district TB and HIV coordinators to make the necessary changes in the facilities. They ensured that the HIV service areas were stocked with TB supplies, such as sputum collection bottles, lab test request forms, TB patient treatment cards, and TB medications; and that HIV and TB providers had equal access to the short message service printers, which are used to quickly transmit results from the lab to the clinic. The only exception was the TB register of cases – we could not get permission from the provincial office to introduce a second TB register for the HIV providers. Thus, HIV providers had to record ‘their’ TB patients on a separate notepad and then transfer the cases at the end of the day into the TB register, which remained at the TB service areas.

### Data collection and analysis

Data collected to assess the proof of concept included routine patient clinical data and endline interviews with health providers and facility mangers. At the end of the study we also met with health providers and facility managers to help interpret study results.

#### Routine patient clinical data

To assess the services provided by health providers in participating facilities, as well as patient outcomes, we followed the records of all patients who were tested for TB during the study period between July 15, 2015 and February 29, 2016 in the three participating facilities, regardless of whether the provider who initiated the test was an HIV or a TB provider, and irrespective of the HIV status of the patient. TB is presumed when the patient exhibits symptoms of TB, however the so called ‘suspect register’ where providers list patients with presumed TB also includes persons in whom the provider wants to exclude TB because of contact with a TB patient. Information about the outcome of the TB test, including dates the samples were taken, tested and reviewed by the laboratory, as well as the test results, were received weekly by each facility directly from the NHLS (NHLS data extracts). For all patients diagnosed with TB, the study team captured additional information about the clinical TB management from the TB register and the patient records (clinic file). To obtain these data a member of the study team visited each of the facilities at least once each month for the 11-month study period. Data sources for the routine patient clinical data are shown in Table 1.

**Table 1:** Data source and data elements

Register of patients with presumed TB (“suspect registers”)	NHLS weekly data extracts	TB registers	Clinic files
Facility name Patient name Date of birth Date sample collected NHLS form number (barcode) Date result received TB result Date TB treatment started	Facility name Patient name Date of birth NHLS test ID Date sample taken Date sample tested Date result reviewed at lab Laboratory name Test type Result Drug sensitivity	Facility name Patient name Date of birth TB registration number Date case registered Provider registering case Type of registration Patient category Classification of disease Date case notified Provider notifying Treatment regimen Date TB treatment started Weight Treatment outcome Date outcome assessment Provider discharging HIV status	Facility name Patient name Date of birth Gender Weight HIV status ID number File number NHLS form number (barcode) Date first contact with clinic Referral status Date first TB test requested Provider requesting first TB test Date on-treatment test requested Provider requesting on-Rx test Date on-Rx test reviewed Provider on-Rx test reviewed Date first dose taken Date last dose IP taken Date first dose CP Date last dose CP Date end-of-Rx test Provider end-of-Rx test reviewed Date X-ray

*Rx* Treatment, *IP* Intensive Phase, *CP* Continuation Phase

To allow for a comparison of how TB patients are managed by HIV providers and TB providers respectively, we also collected information about which service provider (HIV or TB) initiated testing, initiated and monitored TB treatment and/or assessed outcome.

Extensive data cleaning and transformation was undertaken, which included the removal of duplicates and the detection and correction of inconsistent spelling of patient names in various forms and registers. In patients who had been screened and tested for TB on more than one occasion, for example in August 2015 (with a negative TB test) and then again in January 2016, each such ‘episode’ was counted separately. In some cases, during one ‘episode’ more than one TB test was performed. For example, because of an inadequate or indeterminate initial sample which required an additional sample to be tested, or for treatment monitoring of patients who were found to have TB and were initiated on treatment (further tests at the end of the intensive phase and at the end of the continuation phase, to assess treatment outcome during this ‘episode’ of TB). To correctly attribute a test to the respective ‘episode’ required a thorough review of the clinical files.

Because the HIV and TB consultation rooms used different sets of barcodes (to put on testing forms), we were able to distinguish tests initiated by HIV and TB providers, respectively. This allowed us to recognize the occasional switch of provider, as some patients who were screened, tested and initiated on TB treatment by HIV providers were eventually followed up by TB providers, and vice versa.

The database also included patients who had been screened and diagnosed for TB elsewhere, for example by a private general practitioner, and who were only transferred to the study facilities for initiation of TB treatment or to continue TB treatment that had already been initiated.

The clinical database was captured and analysed in Microsoft Excel (2007). By necessity, it included patient names and other identifying information to enable records to be linked. After the database was completed, and the data were cleaned, the clinical data were de-identified to ensure the integrity of the data and maintain patient confidentiality. Analysis included simple frequencies and cross tabulations. Pearson chi-squared (Χ^2^) tests were used to gauge statistical significance at a probability level of 95%. Fisher’s exact tests were used for comparisons when the number of cases was too small for Χ^2^ tests.

#### Interviews with facility health providers and managers

To assess the perceptions, experiences, and preferences of health providers and facility managers regarding the intervention, we conducted anonymous interviews with them in February 2016. A list of all HIV and TB service providers who were on duty at the three intervention clinics on the day of the interview (*n* = 16) as well as supervisors and clinic managers available (*n* = 6) was obtained from the study coordinator. The date for the interviews was set towards the end of the study, during a week the clinics were fully operational, i.e. a week without public or school holidays. The interviews were based on a structured questionnaire and were conducted face-to-face by experienced interviewers, who had been trained on the content and conduct of the interview. The structured questionnaire included mostly closed-ended questions to assess experiences, perceptions and preferences regarding the study intervention. The responses to the closed-ended questions were rated using a 5-point Likert-type scale. Responses to the open-ended questions were noted verbatim. Of the targeted 22 interviewees, 21 agreed to participate and signed the informed consent form (15 service providers and 6 supervisors/facility managers). The data were collected by the interviewers on paper, then entered into Microsoft Excel (2007) for analysis. For the close-ended questions, we analyse frequencies of the single responses as well as measures of central tendency and variability (i.e. mean, standard deviation, Z-score/percentile and coefficient of variance).

## Results

We start by following patient records through the pathway of care, from testing through the completion of treatment, comparing HIV and TB providers. See Additional files 1, 2 and 3 for the complete analysis.

### Screening for TB

A total of 1855 individuals became patients with presumed TB (regardless of HIV status) in the three intervention facilities between July 15, 2015 and February 29, 2016, either because they exhibited TB symptoms or because they came in contact with a sick person. This volume of patients reflects the size of the facilities: 971 patients in clinic 1, 581 in clinic 2, and 303 in clinic 3. Overall, 28.7% of patients for whom we know whether they were screened by HIV or TB provider (*n* = 1524) had been screened by HIV providers. The clinic-specific results differed significantly (*p* < 0.0001) and ranged between 19.8% (clinic 1) and 41.1% (clinic 3). See Additional file 1 for details.

### Testing for TB

Of all patients listed in the register as presumed TB, 86.0% (*n* = 1595) were tested. Table 2 shows the proportion of patients tested for TB in each facility, regardless of HIV status, by type of provider. These are the patients followed in our database. A more detailed analysis is provided in Additional file 1.

**Table 2:** Patients tested for TB by provider

	Clinic 1	Clinic 2	Clinic 3	3 clinics total
	*n* = 862	*n* = 474	*n* = 259	(*n* = 1595)
Tested by HIV provider	127 (14.7%)	139 (29.3%)	88 (34.0%)	354 (22.2%)
Tested by TB provider	544 (63.1%)	251 (53.0%)	129 (49.8%)	924 (57.9%)
Provider not identified	191 (22.2%)	84 (17.7%)	42 (16.2%)	317 (19.9%)

Overall, 75.1% (*n* = 328) and 72.2% (*n* = 785) of patients of HIV and TB providers respectively, were tested with GeneXpert test. The remainder were diagnosed through X-ray or other laboratory tests. Two percent (*n* = 26) of sputum collected were inadequate for testing (unsuccessful). This figure was similar for HIV and TB providers (2.1%, n = 7 vs. 1.9%, *n* = 15).

### TB diagnosis and treatment initiation

Overall, 161 cases of TB were diagnosed in the three clinics during the study period. In eight cases the provider who diagnosed the case could not be determined. Of the 153 cases in which we know the provider, HIV providers diagnosed 57 cases (37.3%) and TB providers 96 cases (62.7%). As Table 3 shows, while in clinics 1 and 2 relatively more cases were diagnosed by TB providers, in clinic 3 HIV providers diagnosed more TB cases. The case-detection rate (percent of patients with TB among those tested for TB) for all three clinics combined was 16.1% for HIV providers and 10.4% for TB providers (*p* = 0.0049), regardless of HIV status. Looking at the clinics separately, case detection rates were higher among HIV providers compared to TB providers (Clinic 1: 22.9% vs. 11.2%, *p* = 0.0005; Clinic 2: 15.1% vs. 12.0%, *p* = 0.3760; Clinic 3: 8.0% vs. 3.9%, *p* = 0.1968). More detailed analysis of cases diagnosed via GeneXpert is available in Additional file 2.

**Table 3:** Patients with confirmed TB by provider

	Clinic 1		Clinic 2		Clinic 3		Unknown provider 3 intervention clinics
	HIV providers	TB providers	HIV providers	TB providers	HIV providers	TB providers	
Tested for TB	127	544	139	251	88	129	318
Identified as having TB	29 (22.8%)	61 (11.2%)	21 (15.1%)	30 (12.0%)	7 (8.0%)	5 (3.9%)	8 (2.5%)
	χ^2^(1) = 11.97, *p* < 0.01		χ^2^(1) = 0.78, ns		χ^2^(1) = 1.67, ns		

All TB cases identified at the intervention clinics during the study period were pulmonary TB; none of the cases were identified as resistant to rifampicin.

### Treatment monitoring and outcome

Overall, 211 cases of TB on treatment were followed during the study period in the three clinics combined. However, 92 of these cases (43.6%) were diagnosed with TB at another facility and transferred in to one of the study sites only to continue and monitor TB treatment. Those transferred cases were followed predominately by TB providers: clinic 1: 44/49 (89.8%), clinic 2: 29/32 (90.6%), clinic 3: 6/11 (54.5%). When presenting results for TB treatment monitoring and outcome we included both, those who were diagnosed in the facility and those who were diagnosed elsewhere and transferred to the facility.

An indicator for the quality of TB treatment monitoring is the proportion of TB patients who had end-of-intensive-phase test and end-of-continuation-phase (full TB treatment) test. HIV providers tested 44.7% of their TB patients at the end of the intensive phase, compared to 55.3% of TB providers (*p* = 0.2549). For the end of treatment we observed higher testing overall (HIV providers: 81.3%, TB providers 75.4%, *p* = 0.7510).

Treatment outcomes appear to be better for patients treated by TB providers, than for those treated by HIV providers, as shown in Table 4, and in more detail in Additional file 3. Two hundred and eleven patients (55 treated by HIV providers and 156 by TB providers) were long enough on treatment to assess the outcome of their intensive phase treatment (at least 60 days, including those who died during that period). Of the patients treated by HIV providers 69.1% (*n* = 38) completed the intensive phase, compared to 79.9% (*n* = 123) of patients treated by TB providers (*p* = 0.1029). Default rate was also higher for HIV providers compare to TB providers (14.5% vs. 3.9%, *p* = 0.0114). On the other hand, fewer patients of HIV providers died during treatment.

**Table 4:** Treatment outcomes by treating provider

	Treatment outcome	HIV providers	TB providers
Intensive phase	N	55	156
	N excluding those still in treatment	55	154
	Completed phase	38 (69.1%)	123 (79.9%)
	Defaulted	8 (14.5%)	6 (3.9%)
	Died	3 (5.5%)	14 (9.1%)
	Transferred or status unknown	6 (10.9%)	11 (7.1%)
Full treatment	N	34	114
	N excluding those still in treatment	33	105
	Cured/completed phase	16 (48.5%)	69 (65.7%)
	Defaulted	10 (30.3%)	10 (9.5%)
	Died	2 (6.1%)	12 (11.4%)
	Transferred or status unknown	5 (15.2%)	14 (13.3%)

When assessing overall treatment outcomes (among patients who started taking treatment at least 180 days before the study ended and who were not taking treatment anymore, or those who died during this period), we found a similar picture. Of patients treated by HIV providers, 16 (48.5%) had completed TB treatment, compared to 69 (65.7%) of those treated by TB providers (*p* = 0.0997). As in the intensive phase, a significantly higher proportion of patients treated by HIV providers defaulted, compared to those treated by TB providers, but fewer of their patients died during treatment. The full analysis is available in Additional file 3.

### Health provider and facility manager perspectives

A total of 21 health providers and facility managers, all from the intervention facilities, were interviewed toward the end of the study in the three intervention facilities – 15 health-care providers, and six supervisors and facility managers. Table 5 shows their responses to the question whether they agreed or disagreed with two positive and two negative statements. Almost all respondents agreed or strongly agreed with both positive statements (*n* = 19, 90.5% each).

**Table 5:** Health provider and facility manager attitudes (*n* = 21)

	Strongly agree	Agree	Neither agree nor disagree	Disagree	Strongly disagree	Mean	Standard Deviation	Z-score to Percentile	Coefficient of Variance
Positive statements									
“HIV providers can collect sputum for TB testing just as well as TB providers”	12 (57.1%)	7 (33.3)	1 (4.8%)	1 (4.8%)	0 (0.0%)	4.4	0.81	0.53 (70.1%)	18%
“Asking HIV providers to provide and monitor TB treatment of their HIV-TB co-infected patients improves TB care and treatment and TB outcome”	9 (42.9%)	10 (47.6%)	1 (4.8%)	1 (4.8%)	0 (0.0%)	4.3	0.78	0.36 (64.2%)	18%
Negative statements									
“Asking HIV providers to provide and monitor TB treatment of their HIV-TB co-infected patients impacts negatively on the quality of HIV care and treatment”	1 (4.8%)	5 (23,8%)	1 (4.8%)	9 (42.9%)	5 (23,8%)	2.4	1.24	−1.26 (10.4%)	51%
“For infection control, it is better for HIV-TB co-infected patients to be treated for HIV in the HIV service and for TB in the TB service”	5 (23,8%)	3 (14.3%)	0 (0.0%)	11 (52.4%)	2 (9.5%)	2.9	1.44	−0.76 (22.4%)	50%

Although the majority disagreed or strongly disagreed with the two negative statements (66.6% and 61.9%, respectively), the responses were mixed, and showed wider variations than the responses to the positive statements.

Health providers and facility managers were also asked an open-ended question about their opinion of the intervention. Most participants positively valued the integration of TB services into HIV care, for example:

*“It [the intervention] helped us a lot because treating HIV and TB separately makes the patients get worse”*

*“It [the intervention] will minimize misdiagnosing of patients”*

*“It [the intervention] was good because it improved TB statistics and the care provided to TB patients. It also reduced stigma to TB patients”*

*“It [the intervention] is good for the patients as well as the facility”*

The few negative comments related to increased workload (three respondents) and additional paperwork (one respondent). One respondent mentioned infection control as a challenge to service integration.

Most health providers and facility managers would recommend replicating the intervention: 20 of 21 respondents (95.2%) said they would recommend that all facilities train and equip HIV providers to *test* their patients for TB; 17 of 21 respondents (81.0%) said they would recommend that all facilities train and equip HIV providers to also *treat* their co-infected patients for TB.

## Discussion

Integration of HIV and TB services is key to improved initiation and outcomes of TB treatment for people living with HIV who are co-infected with TB, and to increased efficiency of the HIV and TB programs in South Africa [25]. This proof of concept study assessed the feasibility and efficacy of integrating HIV and TB services in three public health facilities in South Africa. The primary objective was to assess whether HIV providers can successfully screen their patients for TB, review test results, and manage the TB treatment of their co-infected patients.

### Assessing proof of concept

Our results show that HIV providers can undertake these tasks as well as TB providers. During the study period nearly 30% of the patients who were screened for TB in the intervention clinics were screened by HIV service providers, showing that a relevant proportion of patients to be worked-up for TB, entered the system via HIV service providers. Until now, HIV service providers typically sent patients with presumed TB, i.e. patients who have been found to have symptoms of TB, to the TB service for TB testing and, if applicable, for treatment. Our study shows that HIV service providers can test, and if indicated also treat, patients with TB as well as the TB providers.

The percentage of unsuccessful tests can be an indication for the providers’ technical capacity to make use of GeneXpert testing. HIV and TB providers had a similar proportion of unsuccessful tests (2.1% and 1.9% respectively), at the same level as the national average (1.9%) [28].

It is a well-known phenomenon in TB care in South Africa that not all patients with presumed TB (presence of TB symptoms) progress to testing and treatment, due to challenges of patient adherence, as well as shortcomings on the healthcare system [19]. We found similar percentages of patients ‘lost-to-follow-up’ in the pre-treatment care cascade, from screening to treatment initiation, of patients who were cared for by HIV and TB service providers. It is possible that these patients eventually tested, and if applicable initiated TB treatment, at another facility [29]. As we could see from our own results, patient moving between different facilities is a common practice – nearly half of the patients who started TB treatment at our study sites were transferred in, having been diagnosed with TB at another facility (clinic, hospital or a private general practitioner). Such movements, however, especially in patients who move out without giving or being able to give notice to the provider in advance, are unfortunately difficult to trace [19, 29, 30].

An interesting finding is that HIV providers in our study had more than 50% higher TB detection rate among the patients they tested for TB than the TB service providers. While TB service providers had a detection rate of 10.4%, which is equivalent to the national average [29], the percentage of positive TB tests of HIV service providers was 16.1%. Although the overall TB detection rate varied between clinics, HIV providers had markedly higher detection rates compared to TB providers in all three study sites.

Differences between facilities in the overall detection rate may be explained by differences in the local TB epidemic and the characteristics of living conditions in the catchment areas of the three facilities. Clinic 1, where TB detection rate was highest, is situated in an area where most residential structures are shacks. The area is very crowded, and so TB prevalence can be expected to be higher. In contrast, Clinic 2 and Clinic 3 are located in rural and semi-urban areas, respectively. As most structures are permanent and neighbourhoods less crowded, TB infection rates are expected to be lower. Note, that we reviewed GeneXpert test results from the 6 months prior to the intervention and saw similar proportions of positivity to those we found during the study period.

The different detection rates among people tested for TB between HIV and TB service providers *within* the clinic may be due to differences in the characteristics of the patients typically seen in the two service areas. HIV service providers, for example, tested predominantly symptomatic patients, and only patients who are HIV positive, hence patients with a high pre-test probability of having TB. In contrast, the persons TB providers sent for testing included (i) asymptomatic patients (e.g. patients who had contact with a person with TB (‘household contacts’)); (ii) HIV-negative individuals (individuals known to be HIV positive are predominantly cared for by the HIV service providers); and (iii) patients who are symptomatic because of diseases other than TB (e.g. chronic respiratory disease). Overall, a group of patients in whom the pre-test probability of having TB is very likely lower than in symptomatic patients living with HIV.

Other critical aspects to consider when evaluating the integration of TB and HIV services, especially the integration of full TB services into the scope of work of HIV service providers, are quality of care and treatment outcome. We can assess quality of care by examining the proportion of patients who received microscopic TB tests (Auramine stains) at the end of the intensive phase (‘On-treatment test’) to assess sputum conversion as a prerequisite to scale down to continuation phase treatment; and at the end of the continuation phase (‘End-of-treatment test’) in order to assess cure. Our results show that the overall percentages of on-treatment tests across both service providers were similarly low. HIV service providers had only 44.7% of their patients tested, while TB service providers had 55.3% tested (difference not significant, *p* = 0.2549). Even if considering that not all patients were able to produce a suitable sputum sample anymore, because bronchial secretion had subsided as a result of the treatment, the figures are clearly too low. The situation looks much better with respect to end-of-treatment testing: While 75.4% of the patients treated by TB service providers had an end-of-treatment test, HIV service providers had 81.3% of their patients tested (difference not significant; *p* = 0.6169). These findings indicate that HIV service providers are performing similarly to the TB service providers, regarding monitoring TB treatment. However, improvements, especially regarding on-treatment testing, are urgently needed for all providers.

With respect to TB treatment outcomes, our study revealed that TB service providers performed better than HIV service providers (*p* = 0.0232). The proportion of patients with a successful treatment, i.e. patients who completed treatment without an end-of-treatment test, but with clinical improvement, or patients who had a negative end-of-treatment test and thus could be regarded as cured, was 68.3% for TB service providers and 53.3% for HIV service providers. Irrespective of any differences, completion/cure rates below 80%, are clearly not acceptable, as set by national policy. Several factors account for the overall low treatment success rates in the three study sites. First, high defaulter rates— the defaulter rate among patients treated by HIV service providers was 33.3%, more than 3-fold higher than among patients treated by TB service providers (9.9%, *p* = 0.0024). Second, high mortality rates. Although not statistically significant (*p* = 0.3904), mortality of 11.9% among patients treated by TB service providers was almost double the mortality of 6.7% among patients treated by HIV providers. Third, high percentages of patients transferred out, which affected both services equally (10%).

Our results, therefore, show that HIV providers are as capable as TB providers of screening their HIV-positive patients for TB symptoms, testing the ones with presumed TB, initiating TB treatment in those who are identified as having TB, and monitoring TB treatment. These results demonstrate that HIV service providers are *technically and functionally* capable of integrating TB services into the spectrum of services they provide.

The fact that the overall treatment success rates of both types of providers in our study are poor, and even worse where TB services are provided by HIV service providers, should not be used as an argument to oppose efforts of service integration. Rather, it reveals the limitations of service integration in improving treatment outcomes. The literature shows that service integration definitively contributes to achieving optimal treatment outcomes [18]. However, treatment outcome is not determined only by the quality of services a patient receives at a facility. Factors related to the characteristics of the individual patient, and the population the patient is part of, may contribute as well. For example, the mobility of a patients, which is also partly linked to the responsiveness of the health system to deal with mobile populations and thus to the accessibility of health services in general, or health literacy which may impact on health-seeking behaviour and treatment adherence. Patients who are stably settled residents with good access to a local clinic, and who understand the need to seek healthcare early and adhere to any treatment for the entire course, even though the signs and symptoms of disease have vanished, would certainly benefit from an intervention that increases service quality at the local clinic. This is likely different for patients who only stay temporarily in the area, have difficulty accessing the clinic or services, or present themselves already in more advanced stages of TB. Further studies are required to assess factors that predict treatment outcome at the level of the individual patients and the populations they come from. Our study was not designed to determine this. When results of this study were shared with stakeholders at the provincial and district governments, attendees speculated that the high defaulter rates, especially among HIV patients, may be attributable to the fact that HIV patients in general have high default rates, because of the tiring lifelong treatment which may make them prone to stop taking their medications once they feel better.

### Provider attitudes

The secondary objective of the study was to understand what the integration of TB into HIV services means for the staff and for clinic operations. At the start of the study, some TB providers were opposed to the intervention, and some even tried to sabotage the intervention. For example, we found from the records of the study coordinator, that providers in one facility purposefully misplaced TB forms from the HIV service areas. Such challenges improved over time, as observed when we interviewed providers, supervisors and program managers toward the end of the study period. When asked whether they agreed or disagreed with positive statements, responses were overwhelmingly positive; when asked about negative statements, the responses were mixed, and showed wider variations than the responses to the two positive statements. In other words, responses to the negative statements reveal that although the majority of respondents believed that service integration might *not* negatively affect the quality of HIV services, some concerns remain. Similarly, while the majority said that concerns regarding infection control are *not* an argument against the intervention, a number of respondents had reservations towards the intervention because of infection-control concerns.

These interviews showed almost uniform approval of the intervention, and a general opinion that service integration can improve health outcomes. These sentiments were echoed with stakeholders of all levels (facility, district, province, national) when results of the study were shared with them in dissemination meetings. Yet several critical issues emerged that must be addressed for successful integration of HIV and TB services, including scope of duties, administration, and infection control.

Adding TB testing and treatment to the services rendered by HIV providers means that they learn a new task that may increase their workload, if no adjustments are made, such as allocating more staff. As no additional staff were appointed or shifted to the HIV service as part of the intervention, indeed some staff complained of increased workload. Therefore, HIV and TB service integration should be accompanied by adequate human resource shifts. This is especially true in larger facilities, where staff tend to be more ‘specialized’, such that individual staff members are expected to cover only a certain section of the spectrum of services. This vertical distribution of services also allows for the appointment of less-qualified staff for specific tasks, where they can work under the supervision of a more qualified staff member. This is a common practice in larger facilities, and was the case in Clinic 2, where the TB focal person was not a qualified nurse. Therefore, service integration seems to have a larger impact on the individual service providers in larger facilities. The ‘specialized’ nurse may fear for his/her ‘specialist’ status and need to become competent in other service areas; less qualified staff may fear for their employability. Unavoidably, any change in operational practices can have negative impact on some staff. Key to getting staff support for service integration of HIV and TB services is to clearly communicate plans, address foreseeable positive and negative implications of the intervention, take concerns of the staff seriously, and respond promptly to any unforeseeable developments.

The increase in paperwork, which was criticized by some providers when interviewed, was indeed a concern in this study. It was mainly due to the fact that we could not get permission from the provincial office to introduce a second TB register for the HIV providers. Their concern was that this could result in duplicate records if a patient is registered in one side of the facility, then is treated by a provider from the other side. As a result, HIV providers had to record ‘their’ TB patient information on a separate notepad and then transfer the cases at the end of the day into the TB register, which was kept in the TB service areas. These registers are a critical monitoring and evaluation tool of the National TB-control program. If TB services are rendered at more than one service point in a clinic, each of these service points must be reliably linked. While we could not satisfyingly resolve this issue for this study, we believe that it should be possible to run more than just one register per clinic without affecting the quality of reporting. The use of electronic registers may also resolve this issue.

Infection control was mentioned as a challenge by one provider and emerged as a serious concern during the dissemination meetings, particularly at the district level. Stakeholders were understandably concerned about having symptomatic TB patients treated in the same area as other patients, especially potentially immunocompromised patients with HIV infection. The discussions further revealed that infection control is generally not managed and practiced well at the facilities currently. Therefore, for successful integration of HIV and TB services, infection control must be reviewed and improved across all facilities. Relatively simple infection-control measures, such as open windows or outside or otherwise well-ventilated waiting areas, should be practiced facility wide. Depending on the physical structure of the facility, another infection control measure can be to screen patients as soon as they enter the facility, at the triage or vital signs station, and prioritize those with a cough, or have them wait in a separate area [31].

### Support for HIV and TB service integration

Before HIV and TB services can be integrated on a large scale, there must be support for such a policy at all levels. Stakeholders at the facility, district, provincial, and national levels overwhelmingly approved the intervention. At the facility level, TB providers initially resisted the intervention, but when they realized that HIV and TB service integration will not threaten their positions in the facility they relented, and by the end of the study they showed support for service integration. Support for HIV and TB service integration was evident at the national level, as high-level officials from the National Department of Health supported the study throughout and were excited by the results [25]. Also, officials at the provincial and district levels universally affirmed the integration of TB services into the spectrum of services of HIV providers, and were hoping to implement the service integration in additional facilities.

The ‘Ideal Clinic’ initiative, a complex health intervention which explicitly includes the ‘integrated chronic disease management’ (ICDM), provides an ideal framework for the integration not only of TB and HIV services but also services for other chronic diseases [15]. This concept has been piloted in 2011 and is now being scaled up in South Africa.

### Limitations of the study

The study provides important insights on the feasibility of HIV and TB service integration but has some limitations. First, at the start of intervention implementation not all staff members followed the protocol equally well. We believe this was because they did not see this as part of their normal duties, and some may have feared negative consequences for their job. Through the appointment of local study assistants, regular and ad hoc visits by the study coordinator, repeated training and establishing close and trustful communication lines with facility staff, we were able to minimize non-adherence to the study protocol and procedures and conducted the study as intended.

Second, in order to investigate *how* HIV providers managed TB testing and TB treatment, we utilized routinely captured data from TB screening and case registers, routine data from NHLS, as well as patient-records. Although we believe that data quality overall was good, some information which we intended to use for more detailed analyses was not reliably recorded in the files, such as the presence and duration of TB symptoms. We also identified discrepancies between NHLS data and the TB screening registers and some data quality issues within the NHLS data set (e.g., misspelling of names and incorrectly captured dates of birth). These quality issues occurred only in a fraction of cases, were managed through comprehensive data cleaning, and did not influence our findings.

Third, because the project which funded the study ended, we could assess treatment outcome only on approximately 70% of all the patients whose records we followed. However, as we considered in the final outcome analyses only cases that were long enough on treatment to allow for completion of treatment within the study period, we believe that the essence of our findings regarding outcomes remains unaffected by the shortening of the data collection period.

Finally, this proof of concept study tested the feasibility of the intervention in only three public primary health-care facilities. Therefore, generalizing our findings to other settings should only be done with caution. Nevertheless, we strongly believe that our findings are of great value to the health sector in South Africa, especially to guide the ‘integrated chronic disease management’ which is part of the ‘Ideal Clinic’ programme and a major part of the South African health sector reform. Our findings may also be relevant to other countries with similar settings and a high burden of TB, HIV, and TB/HIV co-infection.

## Conclusions

Service integration, whether actual or functional, is an efficiency solution that can increase the impact of health-services delivery, particularly in resource limited settings. Where population prevalence of HIV and TB are both high, as in South Africa, it is a recognized approach, but one that has proven difficult to implement, both services having evolved at different times and with distinct cultures. Understanding enablers and disablers of service integration is therefore critical to progress against these two deadly diseases. We conclude that HIV providers can successfully test their patients for TB, identify TB cases, and appropriately initiate and monitor TB treatment of their co-infected patients. The majority of service providers and clinic managers clearly approved the intervention. However, the following three aspects require special attention if TB services are offered by HIV providers on a larger scale. First, change of service delivery, unavoidably, affects the work of the service providers involved, and may provoke negative staff behaviour if the consequences provider status or workload are not anticipated, not adequately communicated or not otherwise compensated for. Second, in order to prevent paperwork and incomplete recording of TB cases, any provider involved in the diagnosis of TB at a facility should have easy access to a TB register. Third, integration of TB services into HIV services or any other form of chronic diseases services as suggested for the ‘Ideal Clinic’ require effective infection control measures to be in place. Although there is no doubt that the integration of TB into HIV services improves the quality of care a facility provides, studies are required to further research the effect of service integration on treatment outcomes.

## Additional files

Additional file 1: Screening outcomes. Full analysis of screening outcomes. (PDF 88 kb)

Additional file 2: GenXpert testing outcomes. Full analysis of GenXpert testing outcomes. (PDF 88 kb)

Additional file 3: Treatment outcomes. Full analysis of treatment outcomes. (PDF 85 kb)

Additional file 4: Provider questionnaire. Instrument used to interview providers and program managers. (PDF 154 kb)

## Abbreviations

ART: Antiretroviral treatment
HIV: Human immunodeficiency virus
ICDM: Integrated Chronic Diseases Management
NHLS: National Health Laboratory Service
PEPFAR: President’s Emergency Plan for AIDS Relief
TB: Tuberculosis
UNICEF: United Nations International Children’s Emergency Fund
WHO: World Health Organization
