# Supplementary material for: Improving management of tuberculosis in people living with HIV in South Africa through integration of HIV and tuberculosis services: a proof of concept study
Source: BMC Health Serv Res. 2018 Sep 14;18:711. doi: 10.1186/s12913-018-3524-9 (PMC6137746; doi:10.1186/s12913-018-3524-9)
Supplement: Supplementary file 1 — Screening outcomes. Full analysis of screening outcomes. (PDF 88 kb) [file 12913_2018_3524_MOESM1_ESM.pdf]

Additional file 1: Screening outcome

	Clinic 1		Clinic 2		Clinic 3		Total	
**Service provider**	**HIV**	**TB**	**HIV**	**TB**	**HIV**	**TB**	**HIV**	**TB**
**SCREENING – # screened for TB at the Clinic, total (n)**	971		581		303		1855	
**By provider (n, percentage of number screened for TB with known provider)**	154 (19.8%)	623 (80.2%)	177 (36.2%)	312 (73.8%)	106 (41.1%)	152 (58.9%)	437 (28.7%)	1087 (71.3%)
			X^2 = 149.6373 ***p < 0.00001***					
**Unspecified provider (n, percentage of total number screened for TB)**	194 (20.0%)		92 (15.8%)		45 (14.9%)		331 (17.8%)	
**TESTING – # tested[1] for TB, total (n, percentage of number screened for TB)**	862 (85.7%)		474 (81.6%)		259 (85.5%)		1595 (86.0%)	
			X^2 = 15.6735 ***p = 0.0004***					
**By provider (n, percentage of number screened for TB by provider)**	127 (82.5%)	544 (87.3%)	139 (78.5%)	251 (80.4%)	88 (83.0%)	129 (84.9%)	354 (81.0%)	924 (85.0%)
	X^2 = 2.4673 p = 0.1162		X^2 = 0.2572 p = 0.6120		X^2 = 0.1598 p = 0.6893		X^2 = 3.6801 p = 0.0551	
**Unspecified provider (n, percentage of number screened for TB unspecified provider)**	191 (98.5%)		84 (91.3%)		42 (93.3%)		317 (95.8%)	
**TB CASES – # TB positive, total (n, percentage of total number tested for TB)**	94 (10.9%)		53 (11.2%)		14 (5.4%)		161 (10.1%)	
			X^2 = 7.516 ***p = 0.0233***					
**By provider (n, percentage of number tested for TB by provider)**	29 (22.8%)	61 (11.2%)	21 (15.1%)	30 (12.0%)	7 (8.0%)	5 (3.9%)	57 (16.1%)	96 (10.4%)
	X^2 = 11.9736 ***p = 0.0005***		X^2 = 0.7838 p = 0.3760		X^2 = 1.6658 p = 0.1968		X^2 = 7.9241 ***p = 0.0049***	
**Unspecified provider (n, percentage of number tested for TB unspecified provider)**	4 (2.1%)		2 (2.4%)		2 (4.8%)		8 (2.5%)	

Service provider	Clinic 1 HIV	Clinic 1 TB	Clinic 2 HIV	Clinic 2 TB	Clinic 3 HIV	Clinic 3 TB	Total HIV	Total TB
**REGISTRATION – # registered for treatment, total (n, percentage of total TB cases)**	73 (77.7%)		37 (69.8%)		11 (78.6%)		121 (75.2%)	
			X^2 = 1.2138 p = 0.5450 (p = 0.5267)*					
**By provider (n, percentage of number tested TB positive by provider)**	24 (82.8%)	49 (80.3%)	13 (61.9%)	24 (80.0%)	7 (100%)	4 (80.0%)	44 (77.2%)	77 (80.2%)
	X^2 = 0.0758 p = 0.7830 (p = 1)*		X^2 = 2.031 p = 0.1541 (p = 0.2069)*		p = 0.4167*		X^2 = 0.1966 p = 0.6575 (p = 0.6844)*	
**TREATMENT STARTED – # treatment started, total (n, percentage of total TB cases)**	73 (76.8%)		35 (66.0%)		11 (78.6%)		119 (73.9%)	
			X^2 = 2.5466 p = 0.2799 (p = 0.3092)*					
**By provider (n, percentage of number tested TB positive by provider)**	24 (82.8%)	49 (80.3%)	12 (57.1%)	23 (76.7%)	7 (100%)	4 (80.0%)	43 (75.4%)	76 (79.2%)
	X^2 = 0.0758 p = 0.7830 (p = 0.5138)*		X^2 = 2.187 p = 0.1392 (p = 0.1208)*		p = 0.4167*		X^2 = 0.2876 p = 0.5918 (p = 0.6881)*	
**Started treatment within 2 weeks**	18 (75.0%)	41 (83.7%)	12 (100%)	22 (95.7%)	5 (71.4%)	2 (50.0%)	35 (81.4%)	65 (85.5%)
	X^2 = 0.7819 p = 0.3766 (p = 0.5276)*		p = 1*		p = 0.5758*		X^2 = 0.3493 p = 0.5545 (p = 0.6068)*	
**DIED – No. died before treatment start (n, percentage of TB cases registered)**	0 (0.0%)		1 (2.7%)		0 (0.0%)		1 (0.8%)	
**UNKNOWN – No. unknown TB start (n, percentage of TB cases registered)**	0 (0.0%)		1 (2.7%)		0 (0.0%)		2 (0.8%)	

[1]any laboratory test; *Fisher's Exact Test (two-tailed)
