# Supplementary material for: Improving management of tuberculosis in people living with HIV in South Africa through integration of HIV and tuberculosis services: a proof of concept study
Source: BMC Health Serv Res. 2018 Sep 14;18:711. doi: 10.1186/s12913-018-3524-9 (PMC6137746; doi:10.1186/s12913-018-3524-9)
Supplement: Supplementary file 2 — GenXpert testing outcomes. Full analysis of GenXpert testing outcomes. (PDF 88 kb) [file 12913_2018_3524_MOESM2_ESM.pdf]

Additional file 2: GeneXpert testing outcome

	Clinic 1		Clinic 2		Clinic 3		Total	
	**HIV**	**TB**	**HIV**	**TB**	**HIV**	**TB**	**HIV**	**TB**
**SCREENING – # screened for TB at the Clinic, total (n)**	971		581		303		1855	
**By provider (n, percentage of number screened for TB with known provider)**	154 (19.8%)	623 (80.2%)	177 (36.2%)	312 (73.8%)	106 (41.1%)	152 (58.9%)	437 (28.7%)	1087 (71.3%)
			$X^2 = 149.6373$ ***p < 0.00001***					
**Unspecified provider (n, percentage of total number screened for TB)**	194 (20.0%)		92 (15.8%)		45 (14.9%)		331 (17.8%)	
**TOTAL # cases with GeneXpert test(s) (n, percentage of total number screened for TB)**	659 (67.7%)		402 (69.2%)		237 (78.2%)		1298 (69.9%)	
			$X^2 = 12.0196$ ***p = 0.0025***					
**By provider**	122 (79.2%)	448 (71.9%)	124 (70.1%))	215 (68.9%)	82 (77.4%)	122 (80.3%)	328 (75.1%)	785 (72.2%)
	$X^2 = 3.3767$ p = 0.0661		$X^2 = 0.0698$ p = 0.7917		$X^2 = 0.3184$ p = 0.5826		$X^2 = 1.2765$ p = 0.2585	
**Unspecified provider**	89 (45.9%)		63 (68.5%)		33 (73.3%)		185 (55.9%)	
**POSITIVE**	66 (10.0)		38 (9.5)		11 (4.6)		115 (8.9)	
			$X^2 = 6.4875$ ***p = 0.0390***					
**By provider**	25 (20.5)	40 (8.9)	12 (9.7)	25 (11.6)	7 (8.5)	4 (3.3)	44 (13.4)	69 (8.7)
	$X^2 = 12.6901$ ***p = 0.0004 (p = 0.0006)****		$X^2 = 0.3077$ p = 0.5791 (p= 0.5954)*		$X^2 = 2.6575$ p = 0.1031 (p = 0.1218)*		$X^2 = 5.4244$ ***p = 0.0199***	
**Unspecified provider**	1 (1.5%)		1 (2.6%)		0 (0.0%)		2 (1.7%)	

	Clinic 1		Clinic 2		Clinic 3		Total	
	HIV	TB	HIV	TB	HIV	TB	HIV	TB
**NEGATIVE**	576 (87.4%)		353 (87.8%)		221 (93.2%)		1150 (88.6%)	
			X^2 = 6.2495 ***p = 0.0439***					
**By provider**	93 (76.2%)	399 (89.1%)	107 (86.3%)	184 (85.6%)	75 (91.5%)	114 (93.4%)	275 (83.8%)	697 (88.8%)
	X^2 = 13.3693 ***p = 0.0003***		X^2 = 0.0325 p = 0.8568		X^2 = 0.282 p = 0.5954		X^2 = 5.1200 ***p = 0.0236***	
**Unspecified provider**	84 (94.4%)		62 (98.4%)		32 (97.0%)		178 (96.2%)	
**UNSUCCESSFUL**	13 (2.0%)		9 (2.2%)		4 (1.7%)		26 (2.0%)	
			X^2 = 0.2369 p = 0.8882					
**By provider**	3 (2.5%)	7 (1.6%)	4 (3.2%)	5 (2.3%)	0 (0.0%)	3 (2.5%)	7 (2.1%)	15 (1.9%)
	X^2 = 0.4471 p = 0.5037		X^2 = 0.2466 p = 0.6194		p = 0.2753*		X^2 = 0.0595 p = 0.8072	
**Unspecified provider**	3 (3.4%)		0 (0.0%)		1 (3.0%)		4 (2.2%)	
**UNKNOWN RESULT**	4 (0.6%)		2 (0.5%)		1 (0.4%)		7 (0.5%)	
			X^2 = 0.1302 p = 0.9370					
**By provider**	1 (0.8%)	2 (0.4%)	1 (0.8%)	1 (0.5%)	0 (0.0%)	1 (0.8%)	2 (0.6%)	4 (0.5)
	X^2 = 0.2551 p = 0.6135 (p = 1)*		X^2 = 0.1562 p = 0.6927 (p = 1)*		p = 1*		X^2 = 0.0433 p = 0.8351	
**Unspecified provider**	1 (1.1%)		0 (0.0%)		0 (0.0%)		1 (0.5%)	

* Fisher's Exact Test (two tailed)
