# Supplementary material for: Improving management of tuberculosis in people living with HIV in South Africa through integration of HIV and tuberculosis services: a proof of concept study
Source: BMC Health Serv Res. 2018 Sep 14;18:711. doi: 10.1186/s12913-018-3524-9 (PMC6137746; doi:10.1186/s12913-018-3524-9)
Supplement: Supplementary file 3 — Treatment outcomes. Full analysis of treatment outcomes. (PDF 85 kb) [file 12913_2018_3524_MOESM3_ESM.pdf]

Additional File 3. Treatment Outcome

	Clinic 1		Clinic 2		Clinic 3		Total	
	**HIV**	**TB**	**HIV**	**TB**	**HIV**	**TB**	**HIV**	**TB**
**Total TB cases on treatment for follow-up**	**122**		**67**		**22**		**211**	
**TB cases on Rx - identified by clinic (n, percentage of total TB cases)**	73 (59.8%)		35 (52.2%)		11 (50.0%)		119 (56.4%)	
**By provider (n, percentage of total TB cases by provider**	24 (82.8%)	49 (52.7%)	12 (85.7%)	23 (43.4%)	7 (58.3%)	4 (40.0%)	43 (78.2%)	76 (48.7%)
**TB cases on treatment - transferred-in (n, percentage of total TB cases)**	49 (40.2%)		32 (47.8%)		11 (50.0%)		92 (43.6%)	
**By provider (n, percentage of total TB cases by provider)**	5 (17.2%)	44 (47.3%)	2 (14.3%)	30 (56.6%)	5 (41.7%)	6 (60.0%)	12 (21.8%)	80 (51.3%)
**By provider (n, percentage of total TB cases by clinic)**	29 (23.8%)	93 (76.2%)	14 (20.9%)	53 (79.1%)	12 (54.5%)	10 (45.5%)	55 (26.1%)	156 (73.9%)
			$X^2 = 10.522$ ***p = 0.0052***					
**Outcome Intensive Phase[1]**								
**Still on Intensive phase**	0 (0.0%)	0 (0.0%)	0 (0.0%)	2 (3.8%)	0 (0.0%)	0 (0.0%)	0 (0.0%)	2 (1.3%)
**Unknown (incomplete data)**	1 (3.4%)	4 (4.3%)	2 (14.2%)	2 (3.8%)	0 (0.0%)	0 (0.0%)	3 (5.5%)	6 (3.8%)
**Completed Intensive Phase**	23 (82.1%)	75 (84.3%)	10 (83.3%)	42 (85.7%)	5 (41.7%)	6 (60.0%)	38 (73.1%)	123 (83.1%)
							$X^2 = 2.4667$ $p = 0.1163$	
**Stopped**	0 (0.0%)	0 (0.0%)	0 (0.0%)	0 (0.0%)	0 (0.0%)	0 (0.0%)	0 (0.0%)	0 (0.0%)
**Defaulted**	2 (7.1%)	5 (5.6%)	0 (0.0%)	0 (0.0%)	6 (50.0%)	1 (10.0%)	8 (15.4%)	6 (4.1%)

	Clinic 1		Clinic 2		Clinic 3		Total	
	HIV	TB	HIV	TB	HIV	TB	HIV	TB
							p = 0.0104*	
**Transferred out**	2 (7.1%)	4 (4.5%)	1 (8.3%)	0 (0.0%)	0 (0.0%)	1 (10.0%)	3 (5.8%)	5 (3.4%)
**Died**	1 (3.6%)	5 (5.6%)	1 (8.3%)	7 (14.3%)	1 (8.3%)	2 (20.0%)	3 (5.8%)	14 (9.5%)
							p = 0.5673*	
	p = 0.8040*		p = 0.1953*		p = 0.1412*		X^2 = 8.705 ***p = 0.0335*** (***p = 0.0372)****	
**End-of-Intensive Phase test (completed IP only)**								
**>=30 days and <60 days of IP (n, percentage of cases completed IP)**	11 (47.8%)	48 (64.0%)	4 (40.0%)	19 (45.2%)	2 (40.0%)	1 (16.7%)	17 (44.7%)	68 (55.3%)
	X^2 = 1.9219 p = 0.1656		X^2 = 0.0898 p = 0.7644		X^2 = 0.7486 p = 0.3869 (p = 0.5455)*		X^2 = 1.296 p = 0.2549	
**Outcome Overall treatment[2]**								
**Started TB Rx >=180 days ago (n, percentage of total TB cases)**	93 (76.2%)		41 (61.2%)		14 (63.6%)		148 (70.1%)	
			X^2 = 5.1648 p = 0.0756					
**By provider (n, percentage of total TB cases by provider)**	22 (75.9%)	71 (76.3%)	3 (21.4%)	38 (71.7%)	9 (75.0%)	5 (50.0%)	34 (61.8%)	114 (73.1%)
	X^2 = 0.0028 p = 0.9575		X^2 = 11.785 ***p = 0.0006 (p = 0.0009)****		X^2 = 1.4732 p = 0.2248 (p = 0.3777)*		X^2 = 2.4612 p = 0.1167	
**Still on Continuation phase**	0 (0.0%)	0 (0.0%)	1 (33.3%)	9 (23.1%)	0 (0.0%)	0 (0.0%)	1 (1.5)	9 (7.9%)
**Unknown**	2 (9.1%)	3 (4.2%)	0 (0.0%)	1 (2.6%)	0 (0.0%)	0 (0.0%)	2 (2.9)	4 (3.5%)

	**Clinic 1**		**Clinic 2**		**Clinic 3**		**Total**	
	**HIV**	**TB**	**HIV**	**TB**	**HIV**	**TB**	**HIV**	**TB**
**Completed/cure**	14 (70.0%)	51 (75.0%)	0 (0.0%)	14 (50.0%)	2 (22.2%)	4 (80.0%)	16 (53.3%)	69 (68.3%)
**Stopped**	0 (0.0%)	0 (0.0%)	0 (0.0%)	0 (0.0%)	0 (0.0%)	0 (0.0%)	0 (0.0%)	0 (0.0%)
**Defaulted**	3 (15.0%)	9 (13.2%)	1 (50.0%)	1 (3.6%)	6 (66.6%)	0 (0.0%)	10 (33.3%)	10 (9.9%)
**Transferred out**	2 (10.0%)	4 (5.9%)	0 (0.0%)	6 (21.4%)	1 (11.1%)	0 (0.0%)	3 (10.0%)	10 (9.9%)
**Died**	1 (5.0%)	4 (5.9%)	1 (50.0%)	7 (25.0%)	0 (0.0%)	1 (20.0%)	2 (6.7%)	12 (11.9%)
	$X^2 = 0.493$ p = 0.9204 (p = 0.8640)*		$X^2 = 7.902$ *p = 0.0481* (p = 0.0827)*		$X^2 = 8.193$ ***p = 0.0422 (p = 0.0210)****		$X^2 = 9.513$ ***p = 0.0232 (p = 0.0319)****	
**End-of-Continuation phase test (completed/cure only)**								
**>=60 days of CP (n, percentage of completed/cure)**	11 (78.6%)	45 (88.2%)	-	4 (28.6%)	2 (100%)	3 (75.0%)	13 (81.3%)	52 (75.4%)
	p = 0.3915*		-		p = 1*		p = 0.7510*	

[1] Patients who started TB treatment at least 60 days before the end of the assessment; [2] Patients who started TB treatment at least 180 days before the end of the assessment; * Fisher's Exact test (two tailed)
