# Supplementary material for: Improving management of tuberculosis in people living with HIV in South Africa through integration of HIV and tuberculosis services: a proof of concept study
Source: BMC Health Serv Res. 2018 Sep 14;18:711. doi: 10.1186/s12913-018-3524-9 (PMC6137746; doi:10.1186/s12913-018-3524-9)
Supplement: Supplementary file 4 — Provider questionnaire. Instrument used to interview providers and program managers. (PDF 154 kb) [file 12913_2018_3524_MOESM4_ESM.pdf]

Additional File 4

Provider/program-manager questionnaire

1.	In what year did you begin working for the Department of Health (in any capacity, in any facility)? [If cannot remember exact year – was it less than 5 years ago or more than 5 years ago?]	Year: ___ ___ ___ ___<br>Less than 5 years ago....1<br>More than 5 years ago ...2	
2.	In what year did you begin working in this facility (in any capacity)? [If cannot remember exact year – was it less than 5 years ago or more than 5 years ago?]	Year: ___ ___ ___ ___<br>Less than 5 years ago....1<br>More than 5 years ago ...2	
3.	What is your *primary* responsibility at this facility – do you spend most of your time providing services to patients or managing the facility or supervising staff?	Providing services..........1<br>--------------------------------<br>Managing/supervising 2	→ 6<br>------------<br>Continue
4.	In addition to management/supervision, *in the last two weeks* did you provide any services to a patient, such as examination, ordering tests and/or prescribing medication?	Yes...............................1<br>--------------------------------<br>No ....2<br>Don't remember 8	 ------------<br>→ 30
5.	Were any of these patients being seen for HIV or tuberculosis?	Yes...............................1<br>--------------------------------<br>No ....2<br>Don't remember 8	Continue<br>------------<br>→ 30
**Questions 6-29 are for service providers**			
6.	*In the last two weeks*, did you personally conduct any HIV rapid test (Fingerprick sample) in this facility?	Yes...............................1<br>No ....2<br>Don't remember 8	
7.	*In the last two weeks*, did you personally collect any blood specimens to be sent to the National Health Laboratory Service for any other HIV tests, such as HIV ELISA test, CD4, viral load?	Yes...............................1<br>No ....2<br>Don't remember 8	
8.	*In the last two weeks*, did you personally collect any sputum specimens to be sent to the National Health Laboratory Service for suspected TB or to monitor a TB patient on treatment or retreatment?	Yes...............................1<br>No ....2<br>Don't remember 8	Continue<br>→ 10<br>→ 11
9.	Did you have any difficulties collecting sputum specimens? What difficulty did you have? Any other difficulty? [Mark all mentioned]	Pt could not cough .... A<br>Patient refused.............. B<br>No supplies on hand ..... C<br>Other............................ X<br>Don't remember .... Y<br>No difficulties.................Z	All:<br>→ 11

10	Why did you not collect any sputum specimens? Any other reason? [Mark all mentioned]	Pt could not cough .... A<br>Patient refused .... B<br>No supplies on hand ..... C<br>Not my responsibility ..... D<br>No patients needed TB test .... E<br>Don't know how to collect sputum ....F<br>Referred to another service provider ....F<br>Other .... X<br>Don't remember .... Y	
11	*In the last two weeks*, did you refer any patient to another service provider to collect specimens for any of the following tests:		
	HIV rapid test	[ ] Yes [ ] No [ ]DR	
	Other HIV tests, such as CD4, viral load (blood)	[ ] Yes [ ] No [ ]DR	
	Sputum for suspected TB	[ ] Yes [ ] No [ ]DR	
	Sputum for smear or culture (TB patient on treatment or retreatment)	[ ] Yes [ ] No [ ]DR	
12	*In the last two weeks*, did you receive or review any report from the National Health Laboratory Service for any HIV test (HIV ELISA test, CD4, viral load)?	Yes................................1<br>--------------------------------<br>No ....2<br>Don't remember 8	 ------------<br>→ 15
13	Were the reports (any HIV test [HIV ELISA test, CD4, viral load]) already in the patient's medical record or did you have to go looking for them?	In medical record............1<br>Had to look for it.............2<br>Both ....3<br>Don't remember 8	
14	How were the reports (any HIV test [HIV ELISA test, CD4, viral load]) prepared – on the form delivered by NHLS, on the SMS printer here in the facility, a hand-written note? Anything else? [Mark all mentioned]	NHLS form ....1<br>SMS printer tape ....2<br>Hand-written note...........3<br>Other..............................7<br>Don't remember 8	
15	*In the last two weeks*, did you personally contact the National Health Laboratory Service for any HIV test result (HIV ELISA test, CD4, viral load) because you could not find it at the facility?	Yes................................1<br>--------------------------------<br>No ....2<br>Don't remember 8	 ------------<br>→ 17
16	How did you contact NHLS – did you use a barcode scanner, your personal phone, the facility phone? Anything else? [Mark all mentioned]	Barcode scanner ....1<br>Personal phone ....2<br>Facility phone ....3<br>Other..............................7<br>Don't remember 8	

17	*In the last two weeks*, did you receive or review any report from the National Health Laboratory Service for <u>any TB test</u> (Xpert, smear microscopy/culture)?	Yes................................1<br>--------------------------------<br>No.................................2<br>Don't remember 8	 ------------<br>→ 20
18	Were the reports (<u>any TB test</u> [Xpert, smear microscopy/culture]) already in the patient's medical record or did you have to go looking for them?	In medical record............1<br>Had to look for it............2<br>Both ....3<br>Don't remember 8	
19	How were the reports (<u>any TB test</u> [Xpert, smear microscopy/culture]) prepared – on the form delivered by NHLS, on the SMS printer here in the facility, a hand-written note? Anything else? [Mark all mentioned]	NHLS form ....1<br>SMS printer tape ....2<br>Hand-written note...........3<br>Other.............................7<br>Don't remember 8	
20	*In the last two weeks*, did you personally contact the National Health Laboratory Service for any <u>TB test result</u> (Xpert, smear microscopy/culture) because you could not find it at the facility?	Yes................................1<br>--------------------------------<br>No.................................2<br>Don't remember 8	 ------------<br>→ 22
21	How did you contact NHLS – did you use a barcode scanner, your personal phone, the facility phone? Anything else? [Mark all mentioned]	Barcode scanner............1<br>Personal phone..............2<br>Facility phone.................3<br>Other.............................7<br>Don't remember 8	
22	There are various ways for service providers to get test results from NHLS. If it were totally up to you, which would be your <u>most</u> preferred method: paper report, barcode scanner, cell phone or computer/internet?	Paper report...................1<br>Barcode scanner............2<br>Cell phone......................3<br>Computer/Internet 4	
23	Why is that? (open-ended)		
24	And if it were totally up to you, which would be your <u>least</u> preferred method to get results from NHLS: paper report, barcode scanner, cell phone, or computer/internet?	Paper report...................1<br>Barcode scanner............2<br>Cell phone......................3<br>Computer/Internet 4	
25	Why is that? (open-ended)		
26	**FILTER**. Check Question 3	Service provider ....1<br>Manager/supervisor 2	 → 30
27	*In your current position at this facility*, do you care primarily for HIV patients or for TB patients?	TB patients.....................1<br>----------------------------------<br>HIV patients ....2<br>Both equally ....3<br>Rotating system 4	→29<br>-------------<br><br>Continue
28	How often do you provide or prescribe TB treatment for your HIV patients who are co-infected with TB – would you say rarely, sometimes or almost always?	Rarely ....1<br>Sometimes.....................2<br>Almost always 3	All:<br>→ 40
29	How often do you provide or prescribe HIV treatment for your TB patients who are co-infected with HIV – would you say rarely, sometimes or almost always?	Rarely ....1<br>Sometimes.....................2<br>Almost always 3	All:<br>→ 40

	**Questions 30-39 are for managers and supervisors**		
30	When TB test results come back from NHLS to this facility, who is responsible for getting the form back to the provider who ordered the TB test?	Reception/file clerk.........1<br>Ordering provider ....2<br>Other.............................7	
31	What is the general turn-around time for getting TB results back from NHLS?	Within 24 hours ....1<br>24-48 hours....................2<br>48-72 hours....................3<br>More than 72 hours ....4<br>Don't know/no opinion ..88	
32	Did this facility receive an SMS printer from NHLS as part of the roll-out of GeneXpert testing for TB?	Yes................................1<br>--------------------------------<br>No ....2<br>Don't know 8	 ------------<br>→ 34
33	What is the current status of the SMS printer received from NHLS?	Still here and working .....1<br>Still here but not working 2<br>Sent to another facility....3<br>Don't know 8	
34	*In the last three months*, in your capacity as manager or supervisor, did you personally contact the National Health Laboratory Service for any test result because it could not be found at the facility?	Yes................................1<br>--------------------------------<br>No ....2<br>Don't remember 8	 ------------<br>→ 36
35	How did you contact NHLS – did you use a barcode scanner, your personal phone, the facility phone? Anything else? [Mark all mentioned]	Barcode scanner ....1<br>Personal phone..............2<br>Facility phone.................3<br>Other.............................7<br>Don't remember 8	
36	There are various ways for facilities and providers to receive test results from NHLS. As a manager or supervisor, which method do you feel is <u>most</u> reliable: paper report, barcode scanner, cell phone, or computer/internet?	Paper report...................1<br>Barcode scanner ....2<br>Cell phone......................3<br>Computer/Internet 4	
37	Why is that? (open-ended)		
38	And which method do you feel is <u>least</u> reliable: paper report, barcode scanner, cell phone, or computer/internet?	Paper report...................1<br>Barcode scanner ....2<br>Cell phone......................3<br>Computer/Internet 4	
39	Why is that? (open-ended)		
	**Questions 40-51 are for all respondents**		
40	*In the last 12 months*, did you receive or participate in any training or refresher training in <u>HIV</u> testing and management (by Dept. of Health, NHLS or another accredited institution)?	Yes................................1<br>No ....2<br>Don't remember 8	

<table>
<tr><td>41</td><td>In the last 12 months, did you receive or participate in any training or refresher training in <u>TB</u> testing and management, including collecting sputum specimens (by Dept. of Health, NHLS or another accredited institution)?</td><td>Yes................................1
No.................................2
Don't remember ....8</td><td></td></tr>
<tr><td>42</td><td>This facility provides care and treatment for HIV patients. Which HIV patients should be tested for TB, that is, should have their sputum collected and sent to NHLS? Any others? [Check all mentioned]</td><td>All with cough................ A
All with fever.................. B
All with night sweats...... C
All with weight loss........ D
All not yet on ART (Wellness patients)........ E
All starting on ART ....F
All newly (3 months) on ART..............................G
All HIV patients, regardless of symptoms or status .... H
Other............................. X
Don't know/no opinion... Y</td><td></td></tr>
<tr><td>43</td><td>What percentage of new HIV patients who enroll for care and treatment at this facility are likely to have active TB?</td><td>___ ___ %
Almost none..............775
Almost all 776
Don't know/no opinion 888</td><td></td></tr>
<tr><td>44</td><td colspan="2">On a scale of 1 to 5, where 1 means strongly agree, 3 means neither agree nor disagree, and 5 means strongly disagree, please tell me what you think about the following statements:
<table>
<tr><th></th><th>1
Strongly agree</th><th>2
Agree</th><th>3
Neither</th><th>4
Disagree</th><th>5
Strongly disagree</th></tr>
<tr><td>HIV providers can collect sputum for TB testing just as well as TB providers</td><td></td><td></td><td></td><td></td><td></td></tr>
<tr><td>Asking HIV providers to provide and monitor TB treatment of their HIV-TB co-infected patients impacts negatively on the quality of HIV care and treatment</td><td></td><td></td><td></td><td></td><td></td></tr>
<tr><td>Asking HIV providers to provide and monitor TB treatment of their HIV-TB co-infected patients improves TB care and treatment and TB outcome</td><td></td><td></td><td></td><td></td><td></td></tr>
<tr><td>For infection control, it is better for HIV-TB co-infected patients to be treated for HIV in the HIV service and for TB in the TB service</td><td></td><td></td><td></td><td></td><td></td></tr>
</table></td><td></td></tr>
<tr><td>45</td><td colspan="2">In May and June last year (2015), this facility trained and equipped HIV providers to test their HIV patients for TB and treat their HIV-TB co-infected patients for TB? What are your opinions about this experience? (open-ended)</td><td></td></tr>
</table>

46	Would you recommend that all facilities train and equip HIV providers to test their patients for TB?	Yes................................1<br>No.................................2<br>Don't know/no opinion....8	
47	Why is that? (open-ended)		
48	Would you recommend that all facilities train and equip HIV providers to treat their co-infected patients for TB?	Yes................................1<br>No.................................2<br>Don't know/no opinion....8	
49	Why is that? (open-ended)		
50	Is there anything else about the experience of the last 10 months that you would like to share? (open-ended)		
51	***THANK YOU FOR SPEAKING WITH ME TODAY!***		
